# Diffusion processes modeling in magnetic resonance imaging

**DOI:** 10.1186/s13244-020-00863-w

**Published:** 2020-04-28

**Authors:** Sergey Morozov, Kristina Sergunova, Alexey Petraikin, Ekaterina Akhmad, Stanislav Kivasev, Dmitry Semenov, Ivan Blokhin, Igor Karpov, Anton Vladzymyrskyy, Alexander Morozov

**Affiliations:** 1 Research and Practical Clinical Center of Diagnostics and Telemedicine Technologies, Department of Health Care of Moscow, 28-1, ul. Srednyaya Kalitnikovskaya, Moscow, 109029 Russia; 2 Hospital center of polyclinics AO, 1-3, ul. Bakuninskaya, Moscow, 105005 Russia; 3 Central Institute of Traumatology and Orthopaedics named after N. N. Priorov, 10, ul. Priorova, Moscow, 127299 Russia

**Keywords:** Magnetic resonance imaging, Diffusion-weighted magnetic resonance imaging, Water diffusion, Emulsion, Imaging phantom

## Abstract

**Background:**

The paper covers modern approaches to the evaluation of neoplastic processes with diffusion-weighted imaging (DWI) and proposes a physical model for monitoring the primary quantitative parameters of DWI and quality assurance. Models of hindered and restricted diffusion are studied.

**Material and method:**

To simulate hindered diffusion, we used aqueous solutions of polyvinylpyrrolidone with concentrations of 0 to 70%. We created siloxane-based water-in-oil emulsions that simulate restricted diffusion in the intracellular space. To obtain a high signal on DWI in the broadest range of *b* values, we used silicon oil with high T_2_: cyclomethicone and caprylyl methicone. For quantitative assessment of our phantom, we performed DWI on 1.5T magnetic resonance scanner with various fat suppression techniques. We assessed water-in-oil emulsion as an extracorporeal source signal by simultaneously scanning a patient in whole-body DWI sequence.

**Results:**

We developed phantom with control substances for apparent diffusion coefficient (ADC) measurements ranging from normal tissue to benign and malignant lesions: from 2.29 to 0.28 mm^2^/s. The ADC values of polymer solutions are well relevant to the mono-exponential equation with the mean relative difference of 0.91%.

**Conclusion:**

The phantom can be used to assess the accuracy of the ADC measurements, as well as the effectiveness of fat suppression. The control substances (emulsions) can be used as a body marker for quality assurance in whole-body DWI with a wide range of *b* values.

## Key points

- Presented physical models feature control substances with predefined apparent diffusion coefficients ranging from normal tissue to benign and malignant lesions.
- Aqueous polymer solutions are models of hindered diffusion mathematically described with a mono-exponential equation.
- Water-in-oil emulsions (silicone oils) are models of restricted diffusion.
- Water-in-oil emulsions can be used as an extracorporeal signal source in routine practice.

## Introduction

Currently, diffusion-weighted imaging (DWI) is widely implemented in scanning protocols for various organs and systems, including whole-body magnetic resonance imaging (WB-MRI). Calculated values obtained through the mathematical processing of DWI data, such as apparent diffusion coefficient (ADC map), are used for accurate diagnostics and treatment.

The ADC is a relative value and depends on the equipment characteristics, scan parameters, and image quality [1, 2]. Therefore, ADC values between different regions of interest should be compared only within a single study [3, 4]. Currently, there is no unitary standard for DWI despite a large number of publications and the widespread utilization. Incorrect field uniformity correction may result in incomplete fat suppression, signal summation, and the appearance of artifacts. Also, in some, tissue’s perfusion effects are present. They are associated with the blood flow in the capillary bed and can be conditionally considered as accelerated diffusion of water molecules. The isotropic diffusion restriction associated with the multicomponent environment (macromolecules, cellular structure) is poorly studied and difficult to simulate, although it occupies a significant place in clinical practice. Some publications introduce mathematical models describing the relationship between ADC, signal-to-noise ratio, and other technical characteristics [2].

The paper describes modern approaches for ADC evaluation and proposes a physical model for monitoring the primary quantitative parameters of DWI and quality assurance.

## Materials and methods

### The principles of DWI

DWI is based on a T2-weighted spin-echo pulse sequence, typically with echo-planar imaging (EPI) readout.

**Fig. 1:**
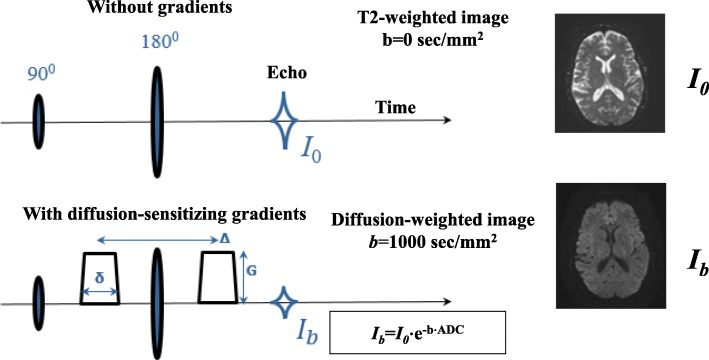
Timing diagram and main characteristics of a diffusion-weighted pulse sequence

Since DWI is a T2-weighted sequence (Fig. 1), signal intensity depends on the repetition time (TR) and echo time (TE) as well as the parameters of the scanned object: proton density (PD) and relaxation times T_1_, T_2_, and ADC:

1 $$ {I}_{\mathrm{DWI}}=k\left(\mathrm{PD}\right)\cdot \left(1-{e}^{-\frac{\mathrm{TR}}{T_1}}\right)\cdot {e}^{-\frac{\mathrm{TE}}{T_2}}\cdot {e}^{-b\cdot \mathrm{ADC}} $$

For most MRI systems, ADC is calculated with the following equation [5]:

2 $$ {\mathrm{ADC}}_{b2-b1}=\ln \left({I}_{b1}/{I}_{b2}\right)/\left(b2-b1\right) $$

This equation does not account for perfusion, which can affect the ADC values.

In most cases, only one *b* value characterizing diffusion gradients can be selected for DWI:

3 $$ b={q}^2{t}_D={q}^2\left(\Delta -\frac{\delta }{3}\right) $$

whereas *t*_*D*_ is the diffusion time, *q*^2^ characterizes gradient pulses, and depends on their amplitude and duration. In a case when the pulse shape is close to rectangular, *q*^*2*^*= (γ G δ)*^*2*^, where γ is the gyromagnetic ratio of hydrogen; G, δ, and Δ are the amplitude, duration of diffusion gradients, and the time interval between them, respectively.

In DWI scan settings, the interval between diffusion-sensitizing gradients (Δ) can be adjusted through the *G* value while the b-factor and *δ* remain constant [6]. For example, with the *b*-factor of 250 s/mm^2^, Δ can be set to 40 ms or 100 ms. Only a slight change in the signal intensity of normal tissue is observed with longer interval Δ and the same *b* value. However, the signal intensity of tissue with restricted diffusion increases by 140% [5]. We consider this a promising direction of future DWI development as it has not yet been widely implemented in clinical practice, but has a high potential for cell size assessment, conversely reflecting the degree of malignancy in some neoplastic processes.

As a result of diffusion-weighted MRI, the radiologist determines the ADC value range. Thus, the *b* value depends on the amplitude of the diffusion-sensitizing gradients and their temporal spacing: different combinations of G, *δ*, and Δ can be set for the constant *b*. In the future, this relationship can be fine-tuned to determine cell size and assess the degree of tissue malignancy.

### The influence of perfusion on diffusion-weighted imaging and apparent diffusion coefficient calculation

As mentioned above, ADC depends on the selected *b* value can represent both perfusion and true diffusion of water molecules [7].

**Fig. 2:**
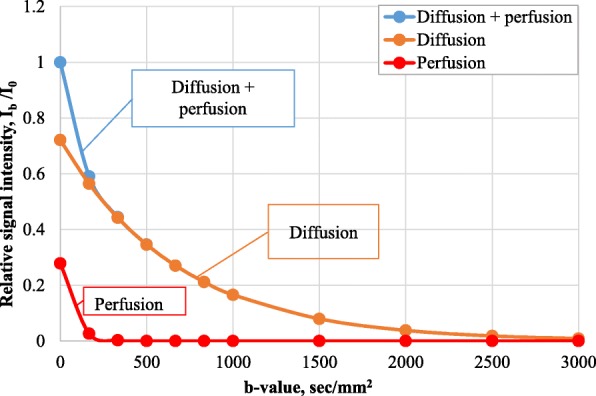
The relationship between relative signal intensity, *b* value, and perfusion (according to data from the [7])

Higher *b* values are associated with a decrease in signal intensity, which was initially defined as mono-exponential, accounting for unlimited diffusion of water molecules only. In the 1980s le Bihan introduced the term “intravoxel incoherent motion” IVIM describing any random movement of water molecules in a voxel [8]. We can evaluate both “fast” diffusion (i.e., microperfusion) and “slow” diffusion (i.e., true diffusion of water molecules) with DWI. The IVIM model is bi-exponential (Fig. 2). This was further expanded with the Kärger model and its modifications reflecting the diffusion restriction between intracellular and intercellular spaces and the mutual exchange of water molecules between them [9].

The decrease in signal intensity with high *b* values corresponds to the anatomical and physiological characteristics of the organ [8]. For example, the liver is characterized by the presence of several types of vessels, sinusoidal capillaries, bile ducts, and a rich lymphatic system, which requires a tri-exponential diffusion model. The mean squared errors of mono-, bi- and tri-exponential models were 44.32, 18.9 and 16.67 respectively.

In routine practice, the ADC values for normal and pathological tissues depend on the *b* values used for the calculation. Perfusion effects will lead to slightly higher ADC values with *b* = 0 s/ mm^2^ and *b* = 1000 s/mm^2^. This decreases the accuracy of ADC calculation and can be corrected with the lowest *b* value of 250 s/mm^2^ or multiple *b* values [1].

### Modeling diffusion processes

In living tissues, water molecules are confined to the intracellular and intercellular spaces with different diffusion patterns. Inside the cell, the movement of water molecules is limited by macromolecules, organelles, and cell membranes. In the intercellular space, outer borders of the cell membranes hinder water molecule diffusion. In normal tissues, the ADC values inside the cell are lower than in the intercellular space. However, an increase in cell density leads to a decrease in intercellular diffusion [9, 10]. Also, there is a continuous mutual exchange of water molecules between the intracellular and extracellular spaces.

**Fig. 3:**
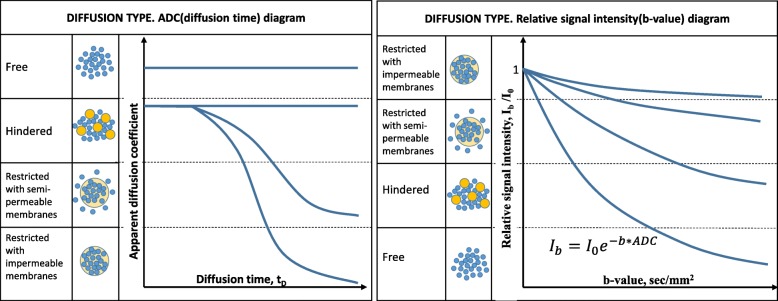
Four diffusion types: non-restricted, hindered, restricted with semi-permeable, and impermeable membranes: **a** Apparent diffusion coefficient dependencies of diffusion time. **b** Relative signal intensity dependencies from *b* value for different diffusion types

The following diffusion models are discussed in the publications: non-restricted (i.e., free), hindered, and restricted with semi-permeable and impermeable membranes (Fig. 3) [11].

In the cases of hindered (slowed) diffusion (*D*_*s*_), ADC values can be used to estimate the average molecular displacement 〈*r*〉 with the Einstein–Smoluchowski relation (three-dimensional version):

4 $$ \left\langle r\right\rangle =\sqrt{6\cdot {t}_D\cdot {D}_s} $$

According to the Einstein-Smoluchowski relation for water molecules simplified as spherical Brownian particles, the diffusion coefficient *D* in three-dimensional space is calculated as:

5 $$ D=\frac{kT}{3\pi \eta d} $$

whereas *η* is the viscosity, *d* is the particle diameter, *k* is the Boltzmann constant, and *T* is the absolute temperature. The equation characterizes molecular movement speed resulting from the Brownian motion.

The diffusion of water molecules inside the cell is restricted (or hindered) by the cell membrane. Therefore, the *D*_*s*_ depends on the diffusion time *t*_*D*_ (equation no. 3) or, in some cases, the Δ (Fig. 3). The 〈*r*〉 value includes information about the cell size. Equal true and the apparent diffusion coefficients will be observed only if the mean square displacement of the water molecule does not exceed the radius of a micelle.

In other words, in a large cell with a semi-permeable membrane, we will register hindered diffusion attributable entirely to the viscosity of the medium. However, with smaller cells or longer *t*_*D*_, the apparent diffusion coefficient will be affected by collisions with the cell membrane, creating a mismatch between apparent and true diffusion and mimicking a slightly hindered diffusion.

Comprehensive DWI quality assurance with different diffusion models, *b* values, and vendor-specific options requires a physical model with predefined varying diffusion restriction levels. Also, the development of such phantom is also vital for WB-MRI as processed images from several radio-frequency coils should reflect the true diffusion as closely as possible and avoid data contamination from the hardware. The phantom needs to generate an extracorporeal signal for comprehensive assessment and differentiation diagnostics.

### Development of a physical model

We developed a comprehensive phantom for quality assurance in DWI (QA-DWI). The phantom contains tubes filled with control substances with predefined ADC values ranging from normal tissue to benign and malignant lesions. Materials with restricted and hindered diffusion were used to simulate intra- and intercellular diffusion.

High or low polymers are usually used to simulate hindered diffusion caused by the collisions of water molecules with macromolecules, cell organelles, or compartments [12]. In the QA-DWI phantom, we used aqueous solutions of polyvinylpyrrolidone (PVP) with concentrations of 0, 10, 20, 30, 40, 50, 60, and 70% by weight.

We created siloxane-based water-in-oil (W/O) emulsions simulate restricted diffusion in the intracellular space. The emulsion consists of water micelles distributed in a fatty medium and surrounded by the emulsifier molecules. The W/O emulsions are reference signal systems representing pathological tissues with low ADC. The optimal substance should have a high signal on DWI images in the broadest range of *b* values, creating an extracorporeal reference signal during the scan.

We used substances with high T_2_, namely cyclomethicone (Cyc-Me) and caprylyl methicone (Cap-Me), to obtain a high signal on diffusion-weighted images.

We measured relaxation times with MR relaxometry. As T_1_ and T_2_ depend, to a small extent, on the magnetic field strength and considering the high availability of 1.5T MR scanners, we chose “Bruker the minispec” relaxometer with operational frequency of 60 MHz, which is close to the resonance frequency of 62.4 MHz for 1.5 T. The T_1_ and T_2_ values were 1070 ± 20 ms and 720 ± 20 for Cyc-Me and 950 ± 20 ms and 174 ± 7 ms for Cap-Me, respectively.

We tested the following emulsions with varying proportions of water/fatty phases and emulsifier (Em):

1:1 Cap-Me:Water (8% Em.),

1:1 Cyc-Me:Water (8% Em.),

2:1 Cap-Me:Water (5% Em.),

2:1 Cap-Me:Water (8% Em.).

We determined emulsion stability by centrifuging and determining isolated phase percentage (fat/water) and selected the emulsions with the highest total and micellar stability. We incorporated silicone oils (cyclomethicone and caprylyl-methicone) into the control substances to simulate fatty tissue.

The reference size of a pathological cell for manufacturing the W/O emulsions was 3–12 μm [5]. According to the dispersion analysis, the size of micelles in the emulsions is 4.8 ± 1.8 μm when diluted with polymethylsiloxane-5 and 4.2 ± 1.6 μm for hexane.

### Scanning parameters

We performed one DWI scan on a 1.5T MR scanner with the following parameters: EPI sequence, TR 2000 ms, TE 120 ms, the number of averages = 1, pixel size 2.0 × 2.0 mm, slice thickness 5.0 mm, echo train length 116, and *b* value 0, 50, 100, 200, ... 1000 s/ mm^2^. Every MRI scan was acquired with two *b* value groups: equal and higher than 0 (i.e., 0 and 50 to 1000 s/ mm^2^) per the technical limits of the scanner. We normalized signal intensity for each *b* value (*I*_*b*_) to the corresponding value *I*_*b*_ = 0. We also qualitatively and quantitatively evaluated fat suppression with the QA-DWI phantom.

We also assessed the phantom as an extracorporeal source signal by simultaneously scanning a patient and elongated sealed W/O emulsion-filled tubes. DWI was performed on another 1.5T MR scanner in WB-MRI mode. Scan options were as follows: whole-body DWI sequence, TR 4600 ms; TE 68 ms; the number of averages = 2; pixel size 2.3 × 2.3 mm; slice thickness 6.0 mm; echo train length 35; and three series of *b* values 0, 5, 10 .... 50; 0, 167, 333, 500, 667, 833, 1000; and 0, 500, 1000 ... 3000 s/mm^2^. In this case, we performed DWI with select *b* values in the following intervals: 0–50, 0–1000, and 0–3000 s/mm^2^.

## Results and discussion

### Phantoms simulating hindered diffusion

**Fig. 4:**
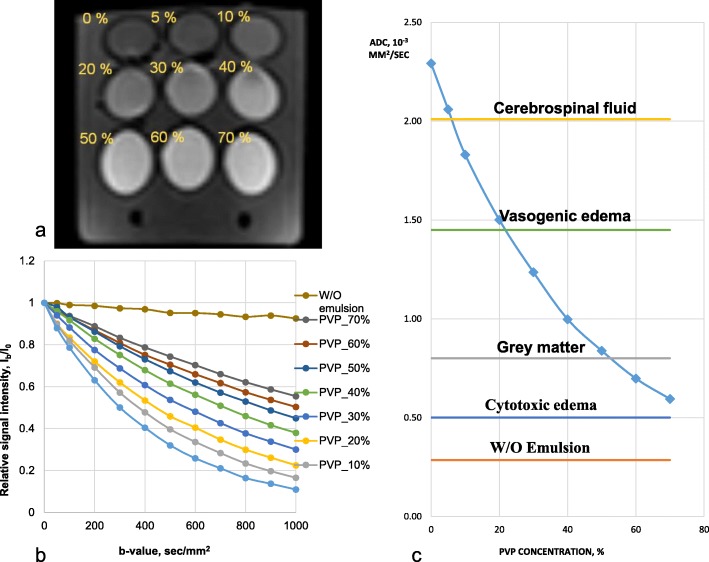
QA-DWI phantom. **a** DWI of the phantom with PVP aqueous solutions (*b* value 500 s/mm^2^). **b**, **c** The dependence graphs of QA-DWI quantitative parameters with **b** being the signal attenuation dependency from *b* value and **c** being the ADC distribution for control substances with values for some normal tissues and pathological conditions

Multi-b DWI with the developed QA-DWI phantom yielded the signal attenuation dependencies (i.e., the ratio of signal intensity with the non-zero *b* value to the signal intensity without the diffusion-sensitizing gradients) for the control substances (Fig. 4a, b). Figure 4c shows the ADC distribution for the aqueous solutions of PVP with varying concentration as well as ADC values of some normal tissues and pathological conditions: cerebrospinal fluid and gray matter [13], vasogenic, and cytotoxic cerebral edema [14].

The ADC value distribution (Fig. 4c) shows that water (PVP concentration 0%) represents the upper ADC limit, the W/O emulsion corresponds to the lower limit, and PVP aqueous solutions—to average values. The total ADC interval accords with 0.28–2.29 mm^2^/s. For a wide range of *b* values, the variability of signal intensity for a 50% aqueous PVP solution averages 5.1%, W/O emulsion—4.6%.

We used aqueous PVP solutions with concentrations ranging from 0 to 70% to simulate hindered diffusion. As per the IVIM theory, such a system should have a nonstationary molecular fraction to be registered on DWI [1]. Namely, the water molecules that initially diffuse freely are only to be hindered by the PVP macromolecules and the increase in solution viscosity. Thus, the signal attenuation of PVP solutions with higher *b* values should correspond to the mono-exponential law. We performed a mono-exponential data approximation (Fig. 4b) via the least-squares method and calculated the *D* coefficient associated with the diffusion of water molecules. A mono-exponential function well approximates the signal attenuation of PVP aqueous solutions with a determination coefficient *R*^2^ ≈ 1.00 (Table 1). The ADC values and the mono-exponential D coefficient are comparable with the mean relative difference of 0.91% (0.05–2.17%).

**Table 1:** Simulated data vs QA-DWI scan results

PVP, %	*D*, *10^−3^ mm^2^/s	ADC, *10^−3^ mm^2^/s	Relative difference, %
0	2.25	2.29	1.77
5	2.04	2.06	0.74
10	1.82	1.83	0.35
20	1.51	1.50	0.52
30	1.23	1.24	0.05
40	0.99	1.00	0.11
50	0.82	0.84	1.48
60	0.70	0.70	1.03
70	0.61	0.59	2.17

*PVP* polyvinylpyrrolidone, *D* approximated coefficient through mono-exponential equation, *ADC* apparent diffusion coefficient through DWI

### Phantoms for fat suppression quality assurance

All DWI sequences incorporate fat suppression techniques to minimize the artifacts due to the displacement of fat and water images. The sequence is nondiagnostic without the fat suppression as the signals from fat and water add up and blur the image along the phase-encoding direction.

**Fig. 5:**
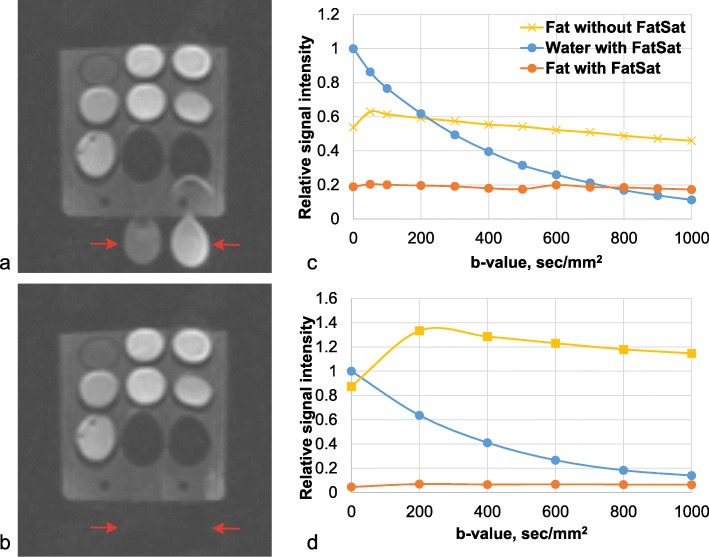
Fat saturation quality assurance with the QA-DWI phantom. **a** DWI (*b* = 1000) with inadequate fat suppression. **b** DWI (*b* = 1000) with adequate fat suppression. Red arrows indicate an extracorporeal signal from the oils. **c**–**d** Signal intensities of fat and water relative to water at a *b*-factor of 0 s/mm^2^ for inadequate (**c**) and adequate (**d**) fat suppression

The QA-DWI phantom allows for precise control of fat suppression. Figure 5 shows adequate and inadequate fat suppression. We performed a qualitative assessment of the fat suppression with Fig. 5 as a reference and the same window level and width. The signal intensities of Cyc-Me and Cap-Me (red arrows) are higher with the faulty fat suppression (Fig. 5a) compared to the complete one (Fig. 5b). Figure 5d shows the relationship between water and fat signal intensity with the *b* value equal to 0 s/mm^2^ in an adequate fat suppression. The signal from fat is suppressed by 18.7 times (Fig. 5d) with adequate suppression and only by 2.9 times with the inadequate one (Fig. 5c).

### Phantoms simulating restricted diffusion

**Fig. 6:**
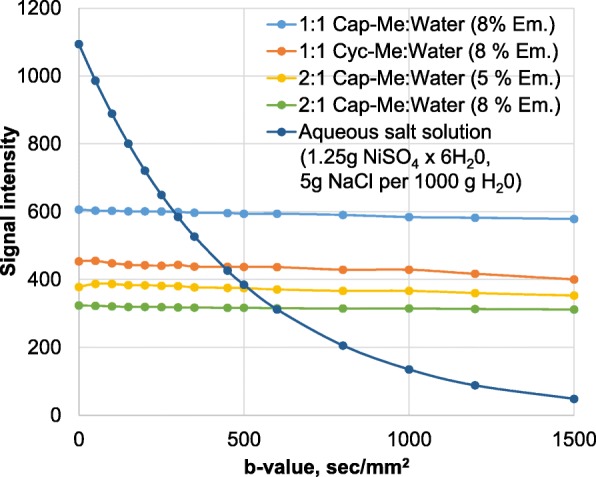
Dependency diagram of DWI signal intensity to *b* value for emulsions with different fat and water phase concentrations

As a result of data processing and comparison of emulsions with different fat and water phase concentrations, we determined that 1:1 Cap-Me: Water (8% Em.) and 1:1 Cyc-Me: Water (8% Em.) had the highest signal intensity within one DWI series (Fig. 6). Also, these W/O emulsions maintained structural and micellar stability as a homogeneous two-phase system for 20 days after manufacture.

### The phantom as an extracorporeal signal source in clinical practice

**Fig. 7:**
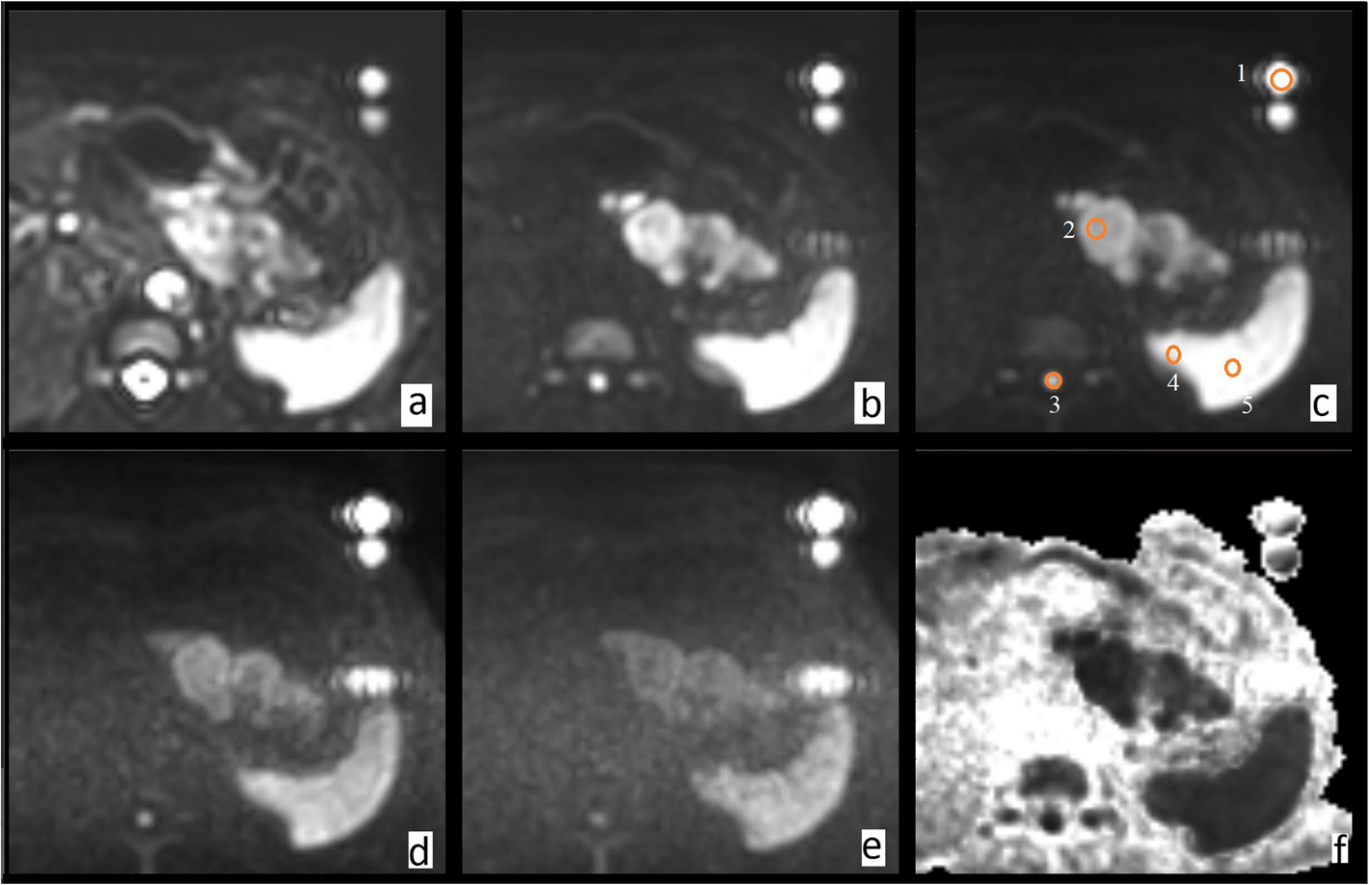
The in vivo DWI with *b*-factor of (**a**) 0 s/mm^2^, (**b**) 500 s/mm^2^, (**c**) 1000 s/mm^2^, (**d**) 2000 s/mm^2^, (**e**) 3000 s/mm^2^, and (**f**) exponential image

We propose the use of QA-DWI phantom as an extracorporeal signal source for DWI-based tumor differential diagnostics, ADC, and artifact correction in WB-MRI. The hermetic tubes are located along the patient’s body. In a test DWI with an extracorporeal signal source, we obtained a series of images (Fig. 7) with following regions of interest: emulsion (1), pancreatic tumor (2), spinal cord (3), kidney (4), and spleen (5). The figure shows isotropic images with *b* value from 0 to 3000 s/mm^2^, as well as an inverted exponential image. We observe artifacts from the oil-based chemical shift and patient movement.

**Fig. 8:**
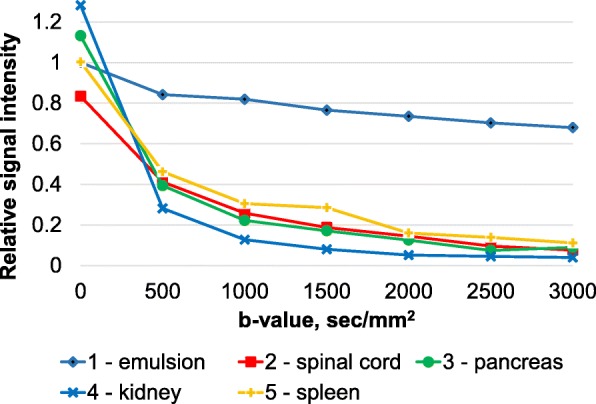
The results of the in vivo study with an extracorporeal signal source: cross plots of the abdominal organs signal intensities relative to the reference signal (emulsion) at increasing *b* value

We measured the signal intensities in these regions for all *b* values and created dependency plots relative to the emulsion simulating the *b* value of 0 s/mm^2^. The emulsions maintained high signal intensity over the entire range of selected *b* values (from 0 to 3000 s/mm^2^) (Fig. 8). The signal from the emulsions allowed us to establish the upper limit of the signal intensity range with a *b*-factor of more than 150 s/mm^2^. The data on the relative signal intensity of emulsion suggests restricted diffusion, which explains only subtle signal attenuation with increasing *b* value. Also, the graph for the kidney demonstrates that several components affect the decay rate, namely a large number of blood vessels, similarly for the liver [8].

The phantom includes aqueous polymer solutions, similar to other devices [12, 15] for modeling diffusion processes and typical ADC range. We also included reverse emulsions for modeling hindered and restricted diffusion, such as Cyc-Me and Cap-Me silicone oils with spectroscopic peaks corresponding to adipose tissue (–CH3/–CH2). The use of these substances allows for a high signal with high *b* values and image quality control in terms of fat suppression.

The prospective use of emulsions lies in modeling pathological processes, including tumor heterogeneity, as well as necrosis and fibrosis, because regions with these processes have pronounced morphological changes and different ADC values [16].

The phantom is suitable for periodic quality control in MRI, as well as multicenter clinical studies with scanners from different manufacturers. Water-in-oil emulsions, as an extracorporeal signal source, increases robustness in comparative assessment and differential diagnostics between benign and malignant lesions. We also scanned our phantom on a 3T unit, demonstrating the feasibility. However, an additional study is required to put it into practice.

Now actively developed digital phantoms that can be used to assess the performance of segmentation under different conditions, such as noise levels or MRI scan protocol [17]. The phantom presented in this work does not allow us to evaluate the modeling of brain structures, because the purpose of its application is the introduction of MRI scanners into the quality control system.

## Conclusion

We reviewed modern approaches to diffusion evaluation with DWI. Models of hindered and restricted diffusion were explored. We developed a phantom containing control substances with predefined apparent diffusion coefficients ranging from normal tissue to benign and malignant lesions. The upper limit was modeled after non-restricted diffusion occurring in water; the lower limit is based on restricted diffusion in W/O emulsions (silicone oils). The average values represent our model of hindered diffusion (aqueous solutions with different PVP concentrations). The QA-DWI phantom can be used to assess the accuracy of the ADC measurements, as well as the effectiveness of fat suppression. The control substances (emulsions) can be used as a body marker for quality assurance in whole-body DWI with a wide range of b-values.

## Data Availability

The datasets used and/or analyzed during the current study are available from the corresponding author on reasonable request.

## Abbreviations

ADC: Apparent diffusion coefficient
Cap-Me: Caprylyl methicone
Cyc-Me: Cyclomethicone
DWI: Diffusion-weighted imaging
EPI: Echo planar imaging
PVP: Polyvinylpyrrolidone
QA: Quality assurance
W/O: Water in oil
WB-MRI: Whole-body magnetic resonance imaging
